# Remote Healthcare During the COVID-19 Pandemic: Findings for Older Adults in 27 European Countries and Israel

**DOI:** 10.3389/fpubh.2022.921379

**Published:** 2022-07-15

**Authors:** Šime Smolić, Nikola Blaževski, Margareta Fabijančić

**Affiliations:** Department of Macroeconomics and Economic Development, Faculty of Economics and Business, University of Zagreb, Zagreb, Croatia

**Keywords:** SHARE Corona Survey, older adults, remote medical consultations, COVID-19, health expenditures, unmet healthcare

## Abstract

The COVID-19 pandemic exacerbated issues regarding access to healthcare for older people, by far the most vulnerable population group. In particular, older adults avoided seeking medical treatment for fear of infection or had their medical treatments postponed or denied by health facilities or health professionals. In response, remote medical services were recognized as an essential adjustment mechanism to maintain the continuity of healthcare provision. Using the SHARE Corona Survey data, we estimate logistic and multilevel regression models for the remote care of 44,152 persons aged 50 and over in 27 European countries and Israel. Our findings suggest that those aged 80+ were the least likely to use remote healthcare. However, women, better educated individuals, older adults who lived in urban areas, those with no financial strain, and active Internet users used remote medical consultations more often. Those who reported poor or fair health status, two or more chronic diseases, or hospitalization in the last 12 months were significantly more likely to use remote healthcare. Furthermore, remote medical consultations were more frequent for those who had their healthcare postponed or went without it due to fear of coronavirus infection. Finally, older adults used remote care more frequently in countries with less healthcare coverage and lower health expenditures. Health systems should prioritize vulnerable groups in maintaining continuity in access to healthcare, despite the availability of remote care. Policymakers should improve telemedicine regulation and offer incentives for providers of remote healthcare services by adapting reimbursement policies. Remote medical care could play an important role in maintaining healthcare access for older adults and increasing health systems' preparedness in future health emergencies.

## Introduction

The unexpected effects of the coronavirus disease (COVID-19) pandemic have led to significant adaptations of health systems in providing healthcare services. One of the adjustment mechanisms includes the more frequent use of telemedicine to maintain continuity in healthcare provision that has been interrupted by epidemic control measures known as stay-at-home orders, lockdowns or social distancing for both COVID-19 patients and those with non-COVID-19 related conditions. The simplest definition of telemedicine is the remote delivery of efficient healthcare services using different innovative information and communication technologies (ICTs) (1). Since the benefits of telemedicine have been recognized in previous public health emergencies, for example, for the Severe acute respiratory syndrome (SARS) or Middle East respiratory syndrome (MERS) (2), this experience has been used, adjusted for the COVID-19 pandemic.

Telemedicine can improve access to healthcare for patients living in remote areas, for example, in rural communities of developing countries who experience limited access to healthcare more often (3). Moreover, it can deploy large numbers of providers rapidly, facilitating the triage and supply clinical services when health facilities cannot meet demands (4). Additionally, it can reduce the risk of complications in individuals with chronic conditions (5) or improve medication adherence for people with chronic diseases (6). Furthermore, remote consultations can help control virus transmission and minimize risk exposure for vulnerable populations (7–9) or improve access to healthcare for patients who fear contagion in health facilities (10, 11).

Altogether, telemedicine has been an efficient, convenient, and affordable healthcare delivery method. It has been used decades before the onset of the COVID-19 pandemic on a relatively small scale, mainly due to professional, technological, or legal barriers (6, 12). However, the COVID-19 pandemic accelerated the implementation and expansion of telehealth and led to a significant change in its perception and role within health systems. The great potential of telemedicine is identified through the more efficient provision of healthcare services for older patients since they put a great deal of pressure on public health spending (13). Besides that, those aged 65 and over were the most vulnerable of the population in the COVID-19 pandemic, both in terms of morbidity and mortality (14–17). Considering that one in five inhabitants of the European Union (EU) in 2020 was 65 years or older (18), the challenges for health systems are unquestionable. Older people with chronic diseases are exposed to a greater risk of adverse long-term consequences of limited access to healthcare (19), and are more likely to experience social isolation and loneliness due to social distancing (20, 21). Consequently, age-friendly remote medical services should, to some extent, mitigate these challenges.

Although we have observed an increase in the reach of telemedicine since the outbreak in European countries, we have little knowledge about older adults who have been using it. This study's primary goal is to better understand the characteristics of older adults that use remote medical care. It explores the relationship between the utilization of remote medical consultations and sociodemographic- and health(care)- related characteristics of older Europeans during the COVID-19 pandemic. To analyze remote healthcare among older adults in the pandemic, we use data collected in the first (June–August 2020) and second (June–August 2021) SHARE Corona Survey (SCS) and supplement them with data from previous SHARE waves. Accordingly, we address the following research questions: (1) Which sociodemographic characteristics are associated with the use of remote medical consultations since the outbreak in Europe and Israel? (e.g., were older adults living in rural areas more likely to use remote medical consultations compared with those living in urban areas?), and (2) Can we relate health system characteristics, for example, organization or financial resources, with older adults' use of remote medical care during the COVID-19 pandemic? The following section presents the international experience of telemedicine and its use among the older population, followed by materials and methods and a section assigned for research results. The final part of the paper provides a discussion and conclusions.

## Telemedicine in the International Context and Its Use Among Older Adults

### International Experience of Telemedicine in the COVID-19 Pandemic

As face-to-face medical consultations have been discouraged, the use and financing of remote consultations (e.g., virtual or phone call visits) have been encouraged since the outbreak. Following the positive experience in China (22, 23), governments in other countries (e.g., Australia, Canada, the United States, and England) relaxed their regulatory frameworks and made their health systems more flexible in response to the COVID-19 pandemic (24, 25). It has been confirmed that countries that invested in telemedicine before the outbreak were more likely to ensure the necessary care for patients (26). Unfortunately, many developing countries where the coverage of telemedicine health services is negligible had not previously invested in telemedicine, and many people are unaware of its practical benefits (12, 27). Bhaskar et al. (28) share perhaps the most detailed study of the state of telemedicine globally before and during the COVID-19 pandemic. They examined telemedicine development and implementation measures in different countries, identified barriers, and proposed actions to integrate telemedicine into public health framework. Many parts of the world (e.g., Africa, the Caribbean, Latin America, and South Asia) either have no national telemedicine frameworks (e.g., in countries like Bangladesh and Mexico); the implementation of telemedicine is hampered due to poorly developed communication technology, conflicts, and war (e.g., in Africa); or its uptake is still relatively slow (e.g., in Argentina) (28). Many European countries recorded a rapid increase in the volume of remote consultations, for example, in primary care (e.g., in Croatia, Malta, Poland, Sweden, and the UK). Remote consultations have increased further in other countries (e.g., Austria, Belgium, Denmark, Estonia, France, Germany, Italy, Luxembourg, and Switzerland) (29). The following examples from several countries illustrate the status of telehealth after the outbreak.

In Germany, Peine et al. (30) investigated the perception of telemedicine. They concluded that medical professionals accept telemedicine, but the many technical and regulatory burdens, especially in university hospitals compared with private health providers, were critical obstacles for additional development. Following the outbreak, France was eager in promoting the use and reimbursement of telemedicine (e.g., for patients with COVID-19 symptoms and those with confirmed COVID-19), while in Italy, telemedicine was not included in the basic package of health services until the end of March 2020 (31). The French healthcare system recognized the telemedicine system before COVID-19—both in terms of regulation and its implementation (28). The limited reach of telemedicine in Italy has been linked to Italian unpreparedness to deal with the COVID-19 health crisis. Here, the telemedicine application was limited mainly due to a lack of implementation and integration of telemedicine services in the national health system from previous years (32). Another example is the UK, where general practitioners were recommended to use video or telephone triage whenever possible to reduce face-to-face contacts (33). Parisien et al. (34) found a positive correlation between COVID-19 disease burden, measured by COVID-19 cases, and the utilization of telehealth services in orthopedic departments across the US. It is essential to mention that even after lifting the lockdown restrictions in April or May 2020, the interest in remote (online) consultations was still sustained (29).

### Use of Telemedicine to Meet Healthcare Needs of Older Adults

Many studies have explored the use of telehealth among older adults during the COVID-19 pandemic and most of them reported positive experiences in terms of patient satisfaction (13, 35–37). An analysis of almost four million consultations at the primary care level between February 17 and May 10, 2020 revealed a more than twofold increase in telephone and electronic video consultations for adults 65 and older in the UK (33). This expansion in terms of video and remote consulting in UK general practice after the COVID-19 outbreak is described as “the biggest evolving natural experiment in general practice in our lifetimes” (38). A study by Gareri et al. (39) found that remote monitoring, either by telephone or video, could protect against the negative consequences of limited access to healthcare for geriatric outpatients. Besides this, Custodero et al. (5) also confirmed telemedicine's usefulness in remotely monitoring the health status of older outpatients. Liu et al. (36) concluded that frail older adults and those without a caregiver to attend assessments with them were less likely to use remote medical care. Bhaskar et al. (13) view the telehealth/telemedicine solutions for the elderly (e.g., home monitoring or telemedicine for those with mental health conditions) as a tool that could lower the burden on public health facilities and an interface that could connect medical specialists with nursing care staff, carers, and patients.

Even though the COVID-19 pandemic prompted many older adults to start using telehealthcare, a “digital divide” between younger and older generations is still evident since older adults are less likely to use or be interested in using telehealthcare (40). This finding has been confirmed in studies before the COVID-19 pandemic; for example, Kontos et al. (41) showed that older men and those with lower education and income in the US were less likely to engage in eHealth activities compared to their counterparts. Additionally, several studies in the early stage of the pandemic in China stress that older people were less prone to use telemedicine applications (9, 42). However, the shift to remote mental healthcare for NHS older adults in the UK did not seem dramatic (43), while in France, the share of older adults (aged 70+) in teleconsultations increased enormously during lockdown (29).

Finally, there are still many unanswered questions about the impact of telemedicine on access to healthcare for older people. Based on earlier works, it seems that this impact is somewhat unclear (35) and the use of telehealth among older people should be viewed from different angles: one where we can see the advantages and another where we observe weaknesses, for example, difficulties to treat patients with cognitive impairment, lack of privacy, technical issues, and so on (12, 13).

## Materials and Methods

### Data and Variables

We gathered publicly available data from the first and the second SHARE Corona Survey (SCS) (*n* = 44,152) (44, 45). SHARE (*The Survey of Health, Ageing and Retirement in Europe*) is a cross-national panel survey that collects microdata on health and the socioeconomic status of individuals aged 50+ in 28 European countries and Israel (46–48). The SCS, which was implemented as a quick response within the SHARE study to understand the effects of the COVID-19 pandemic, asked various questions about key life domains affected by the COVID-19 outbreak, including access to healthcare and the use of remote medical consultations. Data in the first SCS were collected *via* 20–25-min telephone interviews (CATI) from June to August 2020 (49). Additionally, respondents who participated in the first SCS were interviewed again in the second SCS from June to August 2021. Besides the SCS datasets, we use data collected in previous regular SHARE waves (50–57).

### Outcome Variable

The outcome binary variable indicates the use of remote medical consultations since the outbreak. It has been constructed from the following question: “Since the outbreak of Corona, how many remote medical consultations over the phone, computer, or any other electronic means, did you have, if any, with or without video?” This question was asked in the second SCS only, and it captures the use of telehealth in almost a one-and-a-half-year period.

### Explanatory Variables

We use a set of sociodemographic variables (age, gender, living arrangement, education, financial situation, urbanity, and Internet use) and selected health-related variables (e.g., self-reported health, number of chronic illnesses, healthcare use, and unmet healthcare) (see Table 1). The age of respondents is divided into three categories to account for the occupationally active (ages 50–64), young retirees (ages 65–79), and the oldest individuals (age 80+), and the education of respondents is divided into low, medium or high, based on ISCED 2011 classification. The variable “lives with others vs. lives alone” represents the individuals' living arrangements. Simultaneously, the latest available area of residence could be either rural (rural area or village) or urban and is drawn from the regular SHARE waves and the second SCS. Financial situation is a self-reported household financial situation by financial respondents, while the variable “Internet use” indicates if respondents ever used the Internet, for example, e-mailing or searching for information on health-related issues after the outbreak. From the list of health-related variables, we have included: self-rated health (SRH), ranging from poor to excellent and dichotomized into “fair or poor” and “good and better”; the number of chronic conditions (less than two conditions vs. two or more chronic conditions); a dichotomous variable indicating whether the respondent was treated in hospital in the last 12 months; a dummy variable to tell if a respondent has had scheduled medical treatment postponed by a doctor or medical facility; and another one suggesting medical treatments foregone for fear of coronavirus infection. We also employed country controls using a set of country dummies and accounted for health system characteristics: prevailing type of health system financing and organization (Bismarck vs. Beveridge), Universal Health Coverage (UHC) index of service coverage (score below/above 80), health expenditures per capita (in euros adjusted for purchasing power parity) and the number of doctors per 100 thousand inhabitants. Additionally, we excluded respondents in nursing homes (*n* = 385) and interviews collected from proxy respondents (*n* = 1,269). Thus, our final working sample is restricted to 44,152 respondents following the exclusion of randomly missing values for all explanatory variables (the percentages of missing data range from none to a maximum of 2.3% for the variable marking the household economic situation).

**Table 1:** Weighted and unweighted description of the sample micro-level explanatory variables (*n* = 44,152).

		***n***	**Unweighted %**	**Weighted %**
**Sociodemographic variables**				
Age group	50–64	11,324	25.6%	52.1%
	65–79	24,803	56.2%	34.6%
	80+	8,025	18.2%	13.3%
Gender	Women	25,834	58.5%	54.1%
	Men	18,318	41.5%	45.9%
Living alone	Yes	10,991	24.9%	24.6%
	No	33,161	75.1%	75.4%
Education level	Low	14,266	32.3%	32.8%
	Medium	19,330	43.8%	42.4%
	High	10,556	23.9%	24.8%
Area of living	Rural	15,390	34.9%	35.9%
	Urban	28,762	65.1%	64.1%
Economic difficulties	Yes	14,028	31.8%	28.6%
	No	30,124	68.2%	71.4%
Used Internet	Yes	24,361	55.2%	66.2%
	No	19,791	44.8%	33.8%
**Health-related variables**				
SRH	Good and better	26,959	61.1%	65.3%
	Fair or poor	17,193	38.9%	34.8%
Number of chronic illness	≤1 condition	25,958	58.8%	65.4%
	≥2conditions	18,194	41.2%	34.6%
Forwent healthcare	Yes	3,685	8.3%	8.9%
	No	40,467	91.7%	91.1%
Had healthcare postponed	Yes	5,685	12.9%	11.9%
	No	38,467	87.1%	88.1%
Treated in hospital	Yes	8,244	18.7%	19.1%
	No	35,908	81.3%	80.9%

*Weighting done with calibrated cross-sectional individual weights*.

### Data Analysis

Descriptive analyses were carried out to estimate the use of remote medical consultation among people aged 50 and over from the outbreak of the COVID-19 pandemic until the summer of 2021. All categorical variables were reported as count and (un)weighted percentages, while continuous variables were presented as mean. Next, we constructed a multivariable logistic regression model to assess our outcome variable. Each explanatory variable has been previously tested with our outcome variable before being included in the model (*chi*-square and *t*-test; see **Tables 1**–**3**). We apply country controls in our pooled logistic regression model to account for different impacts of the COVID-19 pandemic. We then estimate a multilevel logistic regression model and compare it with the pooled logistic regression results (see **Table 4**). In the next step, we used a multilevel model to explore (country) macro-level factors' effects on the odds of having remote medical consultations during the COVID-19 health crisis. The following section interprets our study results using odds ratios and average marginal effects associated with selected significant micro or macro-level explanatory variables.

## Results

### Micro-Level Explanatory Variables

**Figure 1:**
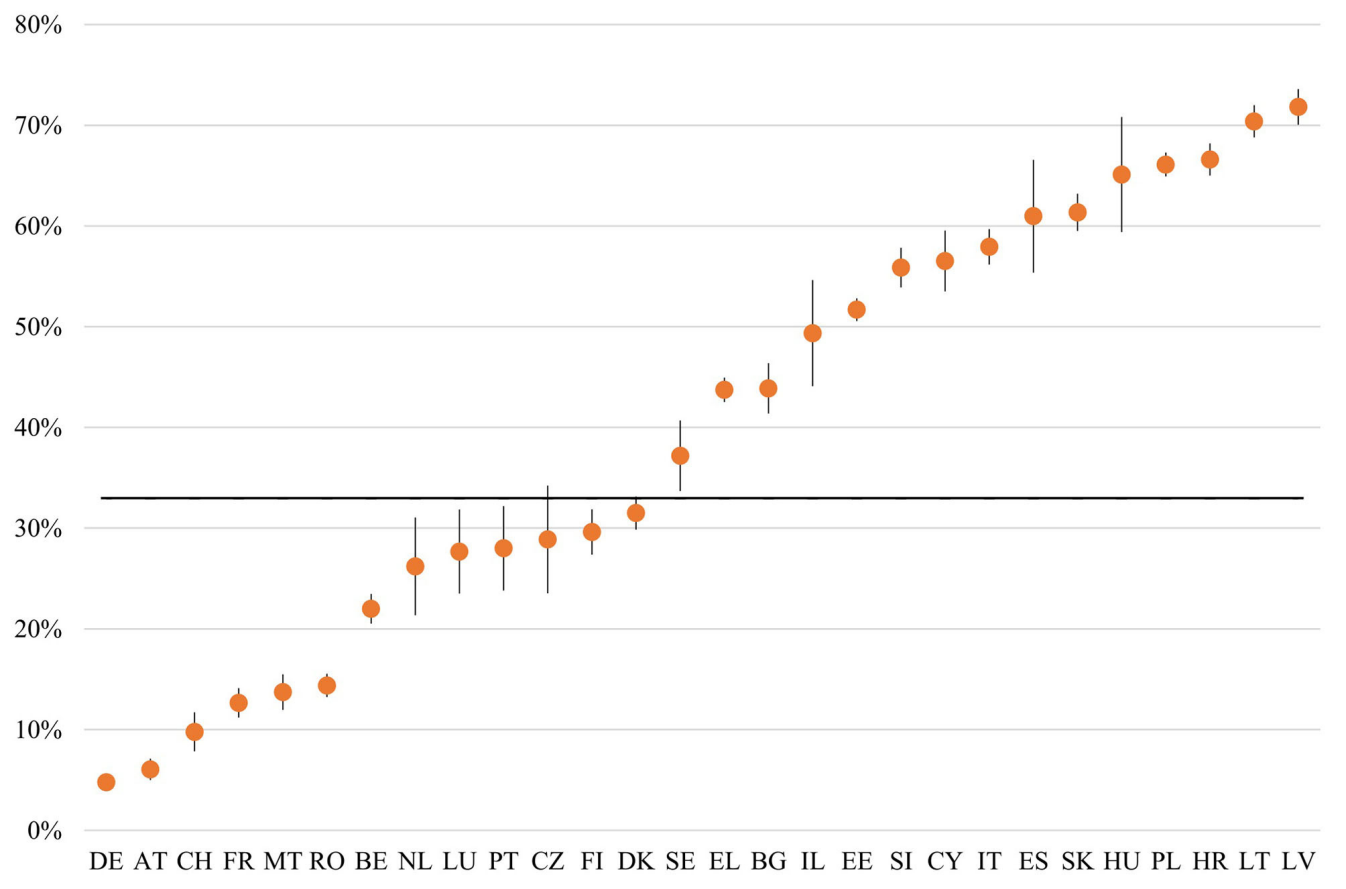
The percentage of respondents aged 50 and over who had remote medical consultations since the outbreak by country (second SCS June–August 2021). Note: Black line represents the sample average–otherwise weighted data with 95% CI.

In our sample from 27 European countries and Israel, almost one in three [33%, 95% CI (31.2, 34.8)] older adults aged 50 and over have had remote medical consultations over the phone, computer, or any other electronic means, with or without video. Figure 1 shows the percentages of older adults who had remote medical consultations by country. Heterogeneity in remote medicine use between European countries is apparent. In Austria and Germany, around 5% and in Latvia and Lithuania, about 70% of those aged 50 and over reported having remote medical consultations since the outbreak.

Table 1 presents descriptive information on the micro-level explanatory variables. Besides the weighted data, we have included unweighted percentages for sample variables^1^.

Table 2 shows the results of testing (*chi*-square tests) of each micro-level explanatory variable independently of the outcome variable (univariate analysis). Use of remote medical consultations after the outbreak was higher among those aged 65–79 (34.1 vs. 32.7% 50–64 and 31.2% 80+), among women (34.8 vs. 30.8% men), and those who lived with others (33.4 vs. 31.6% living alone). It was more common among those with low education (42.5 vs. 27.7% high) and with economic difficulties (45.2 vs. 28.1% without difficulties), older adults in urban areas (36.1 vs. 27.5% in rural), and among those who never used the Internet for e-mailing, searching for information, making purchases, or for any other purpose at least once after the outbreak. Additionally, the use of remote medical consultations is more likely among those with fair or poor overall health (40.5 vs. 29% in good health or better), with two or more chronic diseases (39.7 vs. 29.4% with less than one chronic disease), individuals who have forgone medical treatment due to fear of coronavirus infection (35.3 vs. 32.8%) and those who had their scheduled medical treatment postponed by doctor or health facility (47.5 vs. 31%). Finally, we can see that those treated in the hospital in the last 12 months were significantly more likely to have had remote medical consultations after the outbreak.

**Table 2:** Use of remote medical consultations among people aged 50 and over in 27 European countries and Israel based on the second SCS June–August 2021.

		*n* *	**Weighted %**	**Weighted (95% CI)**	***p*-value for group**
**Sociodemographic variables**					
Age	50–64	4,751	32.7%	29.4%−36.1%	<0.001
	65–79	9,923	34.1%	33.2%−35.1%	
	80+	3,173	31.2%	29.5%−32.9%	
Gender	Women	10,880	34.8%	33.3%−36.4%	<0.001
	Men	6,967	30.8%	27.4%−34.4%	
Living alone	Yes	4,201	31.6%	31.2%−33.7%	<0.001
	No	13,646	33.4%	31.2%−35.7%	
Education level	Low	6,029	42.5%	39.6%−45.4%	<0.001
	Medium	7,772	28.7%	26.9%−30.7%	
	High	4,046	27.7%	22.8%−33.2%	
Area of living	Rural	5,620	27.5%	25.6%−29.5%	<0.001
	Urban	12,227	36.1%	33.6%−38.6%	
Economic difficulties	Yes	7,103	45.2%	42.2%−48.3%	<0.001
	No	10,744	28.1%	26.0%−30.3%	
Used Internet	Yes	9,293	30.6%	28.1%−33.2%	<0.001
	No	8,554	37.6%	35.8%−39.6%	
**Health-related variables**					
SRH	Good and better	9,344	29.0%	26.7%−31.4%	<0.001
	Fair or poor	8,503	40.5%	37.8%−43.3%	
Chronic illness	≤1 condition	8,866	29.4%	26.9%−32.0%	<0.001
	≥2 conditions	8,981	39.7%	37.7%−41.9%	
Forwent healthcare	Yes	1,826	35.3%	30.1%−40.9%	<0.001
	No	16,021	32.8%	30.9%−34.7%	
Had healthcare postponed	Yes	2,937	47.5%	43.5%−51.4%	<0.001
	No	14,910	31.0%	29.1%−33.0%	
Treated in hospital	Yes	3,465	36.2%	33.3%−39.3%	<0.05
	No	14,382	32.2%	30.1%−34.4%	

*Weighting done with calibrated cross-sectional individual weights.*

* *The sum results in 17,847 respondents with the outcome (unweighted)*.

### Macro-Level Explanatory Variables

Table 3 describes the characteristics of macro-level explanatory variables for the countries in the sample. Estimations (not presented here) show that older adults aged 50 and over from countries where the UHC index was below 80 were more likely to use remote medical consultations after the outbreak (52 vs. 31.8% in countries with UHC ≥80; *p* < 0.001). Moreover, the use of remote medical consultations among people aged 50 and over was more prevalent in countries where healthcare is financed dominantly through health insurance contributions (48.7 vs. 37.7% in countries with tax financing; *p*< 0.001). Finally, we have tested the association between per capita health expenditures and health system resources—captured by the number of practicing physicians—and remote medical consultations among older adults. Persons aged 50 and over were less likely to use remote medical consultations in countries where health expenditures per capita were larger. A similar finding appears for the number of practicing physicians.

**Table 3:** Health system characteristics of countries in the sample.

	***n***	**UHC index 2019^**a**^**	**Health expenditures p.c. EUR PPS^**b**^**	**Health system type (financing)^**c**^**	**Practicing doctors per 100 thousand inhabitants^**d**^**
Austria	2,097	82	3,960	Bismarck	531
Germany	1,944	86	4,493	Bismarck	439
Sweden	904	87	3,897	Beveridge	431
Netherlands	667	86	3,956	Bismarck	402
Spain	1,427	86	2,460	Beveridge	440
Italy	2,983	83	2,516	Beveridge	405
France	1,728	84	3,669	Bismarck	317
Denmark	1,528	85	3,797	Beveridge	419
Greece	3,097	78	1,636	Bismarck	349
Switzerland	1,626	87	4,984	Bismarck	434
Belgium	3,228	85	3,828	Bismarck	316
Israel	1,002	84	1,986	Bismarck	330
Czech Republic	1,949	78	2,267	Bismarck	406
Poland	2,485	74	1,516	Bismarck	371
Luxembourg	741	86	3,729	Bismarck	456
Hungary	747	73	1,503	Bismarck	389
Portugal	916	84	2,252	Beveridge	496
Slovenia	2,640	90	2,186	Bismarck	326
Estonia	3,700	78	1,690	Bismarck	346
Croatia	1,636	73	1,358	Bismarck	351
Lithuania	1,155	70	1,725	Bismarck	373
Bulgaria	618	70	1,274	Bismarck	423
Cyprus	530	79	1,862	Beveridge	427
Finland	1,119	83	3,128	Beveridge	464
Latvia	837	72	1,334	Beveridge	326
Malta	652	81	2,754	Beveridge	298
Romania	1,336	71	1,189	Bismarck	237
Slovakia	860	77	1,464	Bismarck	318

*Source: Eurostat (^d^https://ec.europa.eu/eurostat/databrowser/product/view/HLTH_RS_PRS1; ^b^https://ec.europa.eu/eurostat/databrowser/product/view/HLTH_SHA11_HF); World Bank (^d^https://data.worldbank.org/indicator/SH.MED.PHYS.ZS?locations=); OECD (^d^https://data.oecd.org/healthres/doctors.htm); WHO (^a^https://www.who.int/data/gho/data/themes/topics/service-coverage); ^c^This might be also labeled as dominantly Bismarck (Contributions) and Beveridge (Tax or private financed).*

*The Eurostat data for doctors in Malta and Romania are for 2017, data for Denmark and Switzerland are for 2018, data for Finland for 2018 and from World Bank, data for Israel from OECD (converted to EUR PPS), data for Portugal and Greece are from World Bank 2018 and overestimate the absolute number of practicing doctors by around 30%, data for Slovakia are from World Bank 2018 and overestimate the number by about 5%−10%*.

### Empirical Approach and Results

Table 4 presents estimated odds ratios from the pooled logistic regression model with country controls and a multilevel logistic regression model. In the multilevel regression model, we nested individuals in countries and allowed intercepts to vary across countries. The results allowed us to stress the determinants of remote medical consultations among older adults during the COVID-19 pandemic. Then we used multilevel modeling to investigate the effects of country-level variables on the odds of having remote medical consultations during the pandemic. Since macro-level variables exhibit a moderate to strong correlation, they have been added to the models successively.

**Table 4:** Determinants of remote medical consultations among older adults in Europe and Israel after the outbreak of the COVID-19 pandemic.

	**Pooled OR**	**Multilevel OR**
***Sociodemographic variables***	**(odds ratio)**	**(odds ratio)**
**Age**		
50–64	Ref.	Ref.
65–79	0.981	0.980
80+	0.918**	0.917**
**Gender**		
Men	Ref.	Ref.
Women	1.217***	1.217***
**Living alone**		
Yes	Ref.	Ref.
No	1.108***	1.109***
**Education level**		
Low	Ref.	Ref.
Medium	1.057*	1.057*
High	1.161***	1.161***
**Area of living**		
Rural	Ref.	Ref.
Urban	1.149**	1.150**
**Economic difficulties**		
Yes	Ref.	Ref.
No	1.148***	1.150***
**Used Internet**		
Yes	Ref.	Ref.
No	0.754***	0.755***
***Health-related variables***		
**SRH**		
Good and better	Ref.	Ref.
Fair or poor	1.319***	1.319***
**Chronic illness**		
≤1 condition	Ref.	Ref.
≥2 conditions	1.685***	1.685***
**Forwent healthcare**		
Yes	1.449**	1.449**
No	Ref.	Ref.
**Had healthcare postponed**		
Yes	1.666***	1.665**
No	Ref.	Ref.
**Treated in hospital**		
Yes	Ref.	Ref.
No	0.700***	0.701***
Observations	44,152	44,152
Country controls	Yes	No
Multilevel ICC (from the null model)	/	0.268

* *p < 0.1*.

** *p < 0.05.*

*** *p < 0.01*.

**Figure 2:**
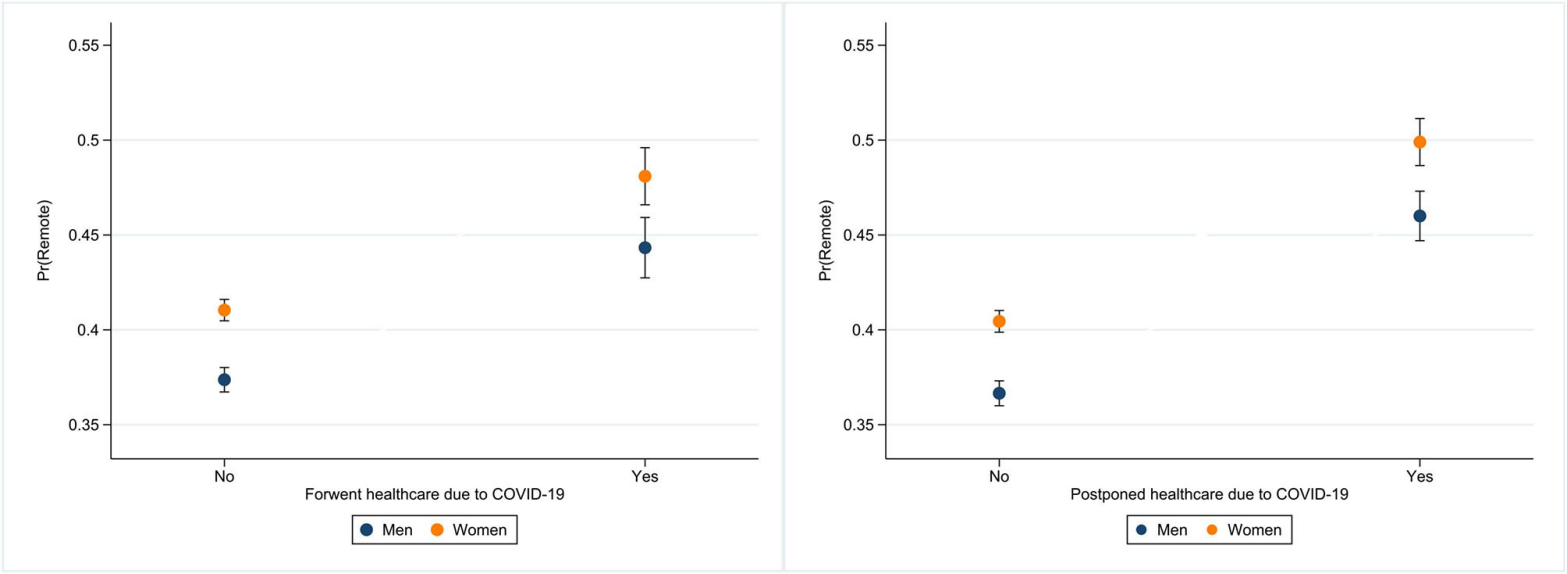
Estimated probabilities of remote medical consultations for men and women and healthcare forgone or postponed.

As expected, the oldest individuals (80+) were the least likely to use remote medical consultations [OR = 0.92, 95% CI (0.85, 0.99)], while on the other hand, women were more likely to use remote care [OR = 1.22, 95% CI (1.16, 1.27)]. The effects of education are noticeable, as more educated older adults aged 50 and over had greater odds of reporting the use of remote care [OR = 1.16, 95% CI (1.09, 1.24)]. Those who lived in urban areas [OR = 1.15, 95% CI (1.09, 1.20)] and who did not experience financial difficulties [OR = 1.15, 95% CI (1.08, 1.21)] were more likely to use remote medical consultations. Being an active user of the Internet increased the odds significantly of using remote medical care among the population aged 50 and over (the odds of remote consultations for this group were around 25% higher). Moreover, health-related variables turned out to be significant predictors of remote care use during the pandemic. Older adults who reported poor or fair SRH [OR = 1.32, 95% CI (1.25, 1.39)] and those with two or more chronic health conditions [OR = 1.69, 95% CI (1.61, 1.77)] were significantly more likely to use remote medical consultations. Further, the odds of reporting remote medical consultation use were 30% lower for older adults who have not been treated in hospital in the last 12 months (before the interview) compared to those who have been hospitalized. Moreover, the odds of using remote healthcare were higher for those who had their scheduled medical treatment(s) postponed (by 67%) or had forgone medical treatment due to fear of coronavirus infection (by 45%). In Figure 2, we show predicted outcome probabilities with 95% confidence intervals for healthcare forgone or postponed, separately for men and women.

**Figure 3:**
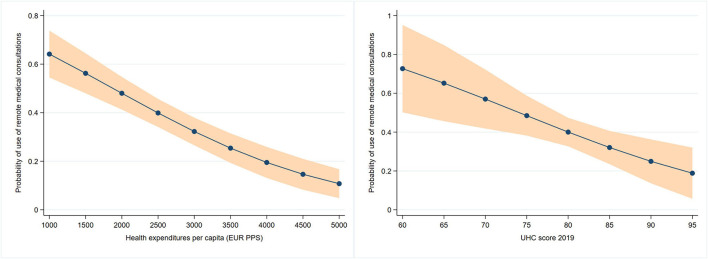
Predictive margins at specified values of macro-level variables.

From Table 5, we can see that respondents from countries with lower UHC index (UHC <80) were 3.4 times more likely to use remote medical consultations compared to respondents in countries with very high UHC index. Further, higher health expenditures per capita were associated with slightly lower odds of using remote care among older adults after the outbreak. We also found greater odds of using remote care for older adults in countries where the tax financing of healthcare prevails and where the average number of practicing physicians is lower, but these results were insignificant. Additionally, in Figure 3 we present predicted outcome probabilities of significant explanatory macro-level variables.

**Table 5:** Country context effects on the use of remote medical consultations among older adults.

	**OR (odds ratio)**
**UHC index**	
≥80	Ref.
<80	3.434***
**Healthcare system**	
“Bismarck”	Ref.
“Beveridge”	1.501
**Health expenditures**	0.999***
**Doctors**	0.996
Observations	44,152
Individual-level controls	Yes

*** *p < 0.01; estimates from multilevel random intercept models*.

## Discussion

This study analyzed the characteristics of individuals aged 50 and over in 27 European countries and Israel who have used remote medical consultations after the COVID-19 outbreak. Since many aspects of medical care have been disrupted by COVID-19, remote medical consultations, at least partially, helped mitigate the backlog in healthcare provision across Europe. When it comes to population groups that have felt this pandemic's most harmful health consequences, older people, especially those with chronic illnesses, can be placed at the top of the vulnerability scale. Many studies have confirmed that the elderly have been the most vulnerable group during the pandemic [see, e.g., (58) or (59)]. Barriers to accessing adequate healthcare triggered many negative consequences for the current health status of older adults. Additionally, delayed medical care utilization and discontinued care—more pronounced during the COVID-19—are expected to adversely affect older adults' health status in the future (60). Here, telemedicine is a promising tool with great potential in reducing unmet healthcare needs (61) and its uptake in some countries after the outbreak was exceptional (33).

One goal of this study was to identify the characteristics of persons aged 50 and over who had remote medical consultations after the outbreak and the other was to explore whether using remote medical consultations was associated with the features of healthcare systems. We found significant differences in the prevalence of remote medical consultations among European countries and Israel, ranging from about 5% in Germany to over 70% in Latvia. We have shown that persons aged 80+, men, and those with poorer socioeconomic statuses were less likely to use remote medical care during the COVID-19 pandemic. Our findings align with those reported in previous studies, such as Kontos et al.'s (41) for the US adult population. Still, this finding is concerning because older individuals could experience the most adverse effects of any population due to missed healthcare during the pandemic.

Furthermore, urbanity was associated with greater odds of remote care use among older Europeans. This finding might be related to the fact that digital infrastructure is better in urban communities, although telemedicine is more often perceived as a facilitator of unmet healthcare in rural areas (3). Our results indicate that more active users of the Internet, and probably those with higher levels of digital literacy, had greater odds of using remote medical consultations. This is in line with conclusions that limited access to broadband Internet and Internet facilities is the main obstacle in the deployment of telemedicine (13) and is a more significant issue, especially in developing countries (7). From this perspective, the message for policymakers is that deeper penetration of digital infrastructure is essential for this segment of healthcare provision to develop.

Health-related variables showed significance in predicting the use of remote medical consultations during the COVID-19 pandemic. Persons aged 50 and over with poorer subjective and objective health statuses were significantly more likely to use remote medical consultations. On the one hand, this could indicate that the COVID-19 pandemic pushed them to switch to this method of accessing healthcare. Still, on the other hand, it could mean that healthcare providers have supplied more remote medical consultations to them because of limitations in the provision of healthcare caused by the pandemic. The latter could suggest the adaptation of healthcare providers and finding new ways of healthcare delivery in the current health crisis. Our following important finding is about the association of unmet healthcare—scheduled medical treatment(s) postponed and medical treatments forgone due to fear of coronavirus infection—and use of remote medical consultations. Generally, the odds of using remote medical care were significantly larger for older adults with unmet healthcare needs. This finding could lead to the conclusion that remote care buffered some unmet healthcare during the COVID-19 pandemic and contributed to continuity in healthcare provision.

Macro-level explanatory variables have been employed to account for health system features that might affect the use of telehealth in different countries [see, e.g., (29)]. Our estimates show that older adults were less likely to use remote medical consultations in countries with a very high UHC index of service coverage. Additionally, we observed lower odds of using remote care among older adults after the outbreak in countries with bigger health expenditures. One could expect that larger densities of practicing physicians were associated with lower prevalences of remote medical consultation, but this variable was insignificant.

To conclude, many countries face problems concerning telehealth to ensure access to regular healthcare. In general, health systems should support both providers and users of telehealth services. However, our findings suggest that health systems should prioritize certain groups for which the continuity in access to healthcare might be a challenge. In particular, these include the oldest individuals (men), people with multiple chronic diseases, those in poor socioeconomic conditions, those living in rural areas, those living alone, and those with poor or no digital skills. Policy strategies may include ongoing support for telehealth, telemedicine regulation, incentives for providers of telehealth services in terms of reimbursement policies, and education about telemedicine. There is evidence that telemedicine can ease the burdens of healthcare delivery in the COVID-19 crisis and improve its access and efficiency (13), but we should be aware that it has limited reach for certain patients who have inadequate resources and access to telemedicine (62). Nevertheless, its importance has already been recognized in some countries (e.g., Denmark), which have incorporated telemedicine into their digitization strategies (29). In addition, telehealth is also of great importance to the European Commission (63), especially in addressing the difficulties in accessing medical services and improving patient outcomes and health system efficiency (64). As the world's population becomes an aging one and we learn to live with COVID-19, remote medical care is a crucial healthcare method that can be accessed safely by older people and ease burdens on healthcare systems.

Further research should also focus on methods that could improve telemedicine penetration and acceptance among at-risk groups in the general population. Inequitable access to telemedicine should be addressed with the expansion of broadband Internet infrastructure and related communication technology, including education and knowledge sharing among both patients (and their families) and medical staff (62, 65). Telemedicine implementation should be accompanied by the harmonization of existing and the introduction of the new regulations to ensure service quality standards and the protection of patient data and privacy. Policymakers should raise awareness of telemedicine benefits for both patients and the healthcare workforce; as already mentioned, telemedicine can act as a link between medical specialists, local nursing home/caring staff, and patients (13). Telemedicine uptake and implementation also need to be accompanied by adequate financial support (e.g., reimbursement), with special emphasis on making it available to at-risk groups—older adults and especially those in poor health and poor socioeconomic conditions.

### Limitations

This study has several limitations. First, many variables are self-reported, and self-reporting bias cannot be disregarded. Second, differences in remote medical consultations usage might result from cross-cultural differences in different countries or other unobserved health system features. Third, the study's design did not permit us to differentiate between various kinds of remote medical consultations after the outbreak and we considered all kinds of remote consultations.

## Data Availability Statement

Publicly available datasets were analyzed in this study. This data can be found at: http://www.share-project.org/data-documentation/share-data-releases.html. All data used in our study are available free of charge to all scientific users world-wide after individual registration (http://www.share-project.org/data-access/user-registration.html). SHARE data are DOI registered datasets. Each wave and each release is assigned a persistent DOI. In our article we use SHARE data from Waves 1, 2, 3, 4, 5, 6, 7, 8 and 9 (DOIs: 10.6103/SHARE.w1.800, 10.6103/SHARE.w2.800, 10.6103/SHARE.w3.800, 10.6103/SHARE.w4.800, 10.6103/SHARE.w5.800, 10.6103/SHARE.w6.800, 10.6103/SHARE.w7.800, 10.6103/SHARE.w8ca.800, 10.6103/SHARE.w8.800, and 10.6103/SHARE.w9ca.800).

## Ethics Statement

The studies involving human participants were reviewed and approved by Ethics Committee of the University of Mannheim and Ethics Council of the Max Planck Society. For more details see: http://www.share-project.org/fileadmin/pdf_documentation/SHARE_ethics_approvals.pdf. The patients/participants provided their written informed consent to participate in this study.

## Author Contributions

ŠS contributed to the conceptualization, methods, implications, and wrote the first draft of the manuscript. NB organized the database. ŠS, NB, and MF contributed equally in performing the statistical analyses. NB and MF wrote sections of the manuscript. All authors contributed to manuscript revision and read and approved the submitted version.

## Funding

This work was supported by the EU Horizon 2020 SHARE-COVID19 project [grant number 101015924]. The SHARE data collection has been funded by the European Commission, DG RTD through FP5 (QLK6-CT-2001-00360), FP6 (SHARE-I3: RII-CT-2006-062193, COMPARE: CIT5-CT-2005-028857, and SHARELIFE: CIT4-CT-2006-028812), FP7 (SHARE-PREP: GA N°211909, SHARE-LEAP: GA N°227822, SHARE M4: GA N°261982, and DASISH: GA N°283646), Horizon 2020 (SHARE-DEV3: GA N°676536, SHARE-COHESION: GA N°870628, SERISS: GA N°654221, and SSHOC: GA N°823782) and by DG Employment, Social Affairs & Inclusion through VS 2015/0193, VS 2016/0135, VS 2018/0285, VS 2019/0332, and VS 2020/0313. Additional funding from the German Ministry of Education and Research, the Max Planck Society for the Advancement of Science, the US National Institute on Aging (U01_AG09740-13S2, P01_AG005842, P01_AG08291, P30_AG12815, R21_AG025169, Y1-AG-4553-01, IAG_BSR06-11, OGHA_04-064, HHSN271201300071C, RAG052527A) and from various national funding sources is gratefully acknowledged (see www.share-project.org).

## Conflict of Interest

The authors declare that the research was conducted in the absence of any commercial or financial relationships that could be construed as a potential conflict of interest.

## Publisher's Note

All claims expressed in this article are solely those of the authors and do not necessarily represent those of their affiliated organizations, or those of the publisher, the editors and the reviewers. Any product that may be evaluated in this article, or claim that may be made by its manufacturer, is not guaranteed or endorsed by the publisher.

## Footnotes

^1^More details about the weighting procedure in SHARE study is available at http://www.share-project.org/faqs/7-weights.html.
